# Efficacy of different low-level laser therapy sessions in the management of masseter muscle trigger points

**DOI:** 10.1186/s12903-024-04780-y

**Published:** 2024-09-26

**Authors:** Nermine Ramadan Mahmoud, Wessam Ibrahim Shehab, Amany Ahmed AlAraby, Yasser Fekry Habaka

**Affiliations:** 1 Oral and maxillofacial surgery Department, Faculty of Dentistry, October 6th University, 6th of October City, Giza Governorate Egypt; 2 Oral Medicine, Periodontology & Oral Radiology Department, Faculty of Dentistry, October 6th University, 6th of October City, Giza Governorate Egypt

**Keywords:** Myofascial pain syndrome, LLLT, Trigger point, Masseter

## Abstract

**Background:**

Low-level laser therapy (LLLT) is one of the recent treatment modalities for myofascial pain dysfunction syndrome with trigger points. The objective of the present study was to examine the impact of varying LLLT sessions on the treatment of masseter muscle trigger points.

**Methods:**

90 patients diagnosed with orofacial pain and trigger points in the masseter muscle for at least 6 months were selected and divided into 3 groups (*n* = 30) based on the number of LLLT sessions provided to patients. Patients in Group I received one session/per week, group II received two sessions/per week, and Group III received three sessions/per week. The sessions continued for 4 weeks, evaluations of pain levels, maximum mouth opening (MMO), and quality of life were conducted before and after the procedure at 1, 2, 3, 4, and 8 weeks.

**Results:**

The pain scores exhibited a highly statistically significant difference among the three groups (*p* < 0.001) over the 8-week study period. MMO was statistically significantly different between groups at week 4 and week 8. The Oral Health Impact Profile-14 (OHIP-14) score was statistically significant difference between groups at week 8. The time showed a highly significant effect on the study outcomes within each group.

**Conclusion:**

Increased the number of LLLT sessions reduced the pain improved the MMO, and subsequently improved the quality of life.

**ClinicalTrials.gov ID:**

NCT06327204 - retrospectively registered.

## Introduction

One of the most frequent causes of non-odontogenic pain in the head and neck area is myofascial pain dysfunction syndrome, affecting approximately 40–60% of adults [1–3]. A clinical indicator of myofascial dysfunction syndrome is the presence of trigger points. It appears as a confined, taut band within muscle fibers. This condition adversely affects the patient’s quality of life through pain, loss of function, sleep difficulties, and taut band characterized by localized taut bands within muscle fibers. loss of function, sleep disturbances, and taut bands [2, 3]. Trigger sites also alter muscle composition and function, potentially leading to condylar regeneration and bone remodeling [4–6].

Various treatment modalities have been introduced to alleviate discomfort and restore function by releasing the trigger point inside the masticatory muscles. These therapeutic approaches encompass manual therapy, LLLT, splint therapy, dry needling, as well as injections of corticosteroids, magnesium sulfate, local anesthetic, botulinum toxins, and physiologic saline [4, 7, 8].

In the last few years, a growing interest in LLLT as a conservative treatment modality was reported with minimal patient discomfort, This effect can be attributed to the enhancement of endogenous opioid release, activation of tissue repair and cellular respiration, increased vasodilation, and alleviating pain [9–11].

Treatment of trigger point-related myofascial pain dysfunction syndrome with laser therapy was recently the focus of many studies. According to several studies LLLT was superior to a placebo [9–11]. A recent systematic review and meta-analysis reported that healthcare professionals were unable to reach definitive decisions regarding the appropriate dosage due to a lack of data [12]. The present study aimed to investigate the effect of varying the number of LLLT sessions on pain levels, MMO, and overall quality of life in patients diagnosed with masseter muscle trigger points. The null hypothesis was that increasing the number of LLLT treatment sessions would not be associated with improved pain levels, MMO and OHIP-14.

## Material & method

The current study was conducted between March 2023 and December 2023 at the Faculty of Dentistry, October 6 University, Giza, Egypt. The study was approved by the Faculty of Dentistry Research Ethics Committee (approval Number: RECO6U/18-2023) and retrospectively registered in the ClinicalTrials.gov database on 20/3/2024. (Identifier ID: NCT06327204).

**Fig. 1:**
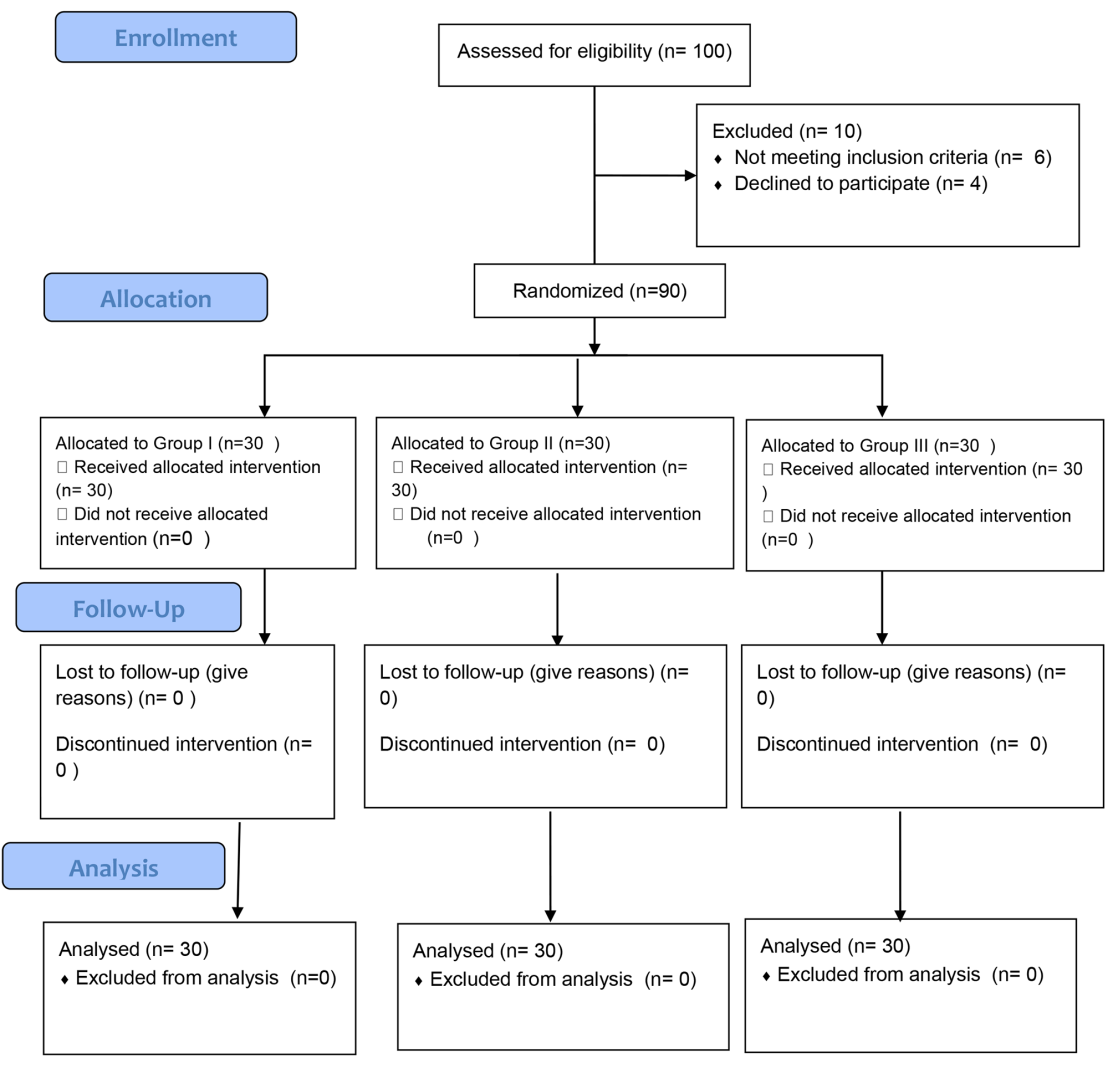
Consort flow chart

Before the trial commenced, informed consent was obtained from each patient to share clinical data and images for research purposes. The study adhered to the Declaration of Helsinki [13] and the CONSORT criteria (2012) for reporting. (Fig. 1) [14] In the current study, an unstratified random block design (block sizes 2, 4, and 6) was employed to ensure balance in the patient number assigned in each group.

A sample calculation was computed based on data from a previous study showed that a total of 63 patients would need to be included for a study power of 80% at an alpha of 0.05. However, this study included a total of 90 patients (30 in each group) to account for any possible dropouts [15]. sample calculation, based on data from a previous study, indicated that 63 patients would be needed for a study power of 80% at an alpha of 0.05. However, this study included a total of 90 patients (30 in each group) to accommodate any potential dropouts.”

A total of 90 patients suffering from orofacial pain and have masseter muscle trigger points for 6 months minimum. The selected cases have the following inclusion criteria: (1) cases with myofascial pain that were diagnosed according to DC/TMD criteria [16] (2) trigger points in the masseter muscle; and (3) no invasive procedures of the masseter muscle before (4) patient with limited mouth opening while the exclusion criteria were (1) painful conditions at orofacial region; (2) Medical conditions affecting the masticatory system function as rheumatoid arthritis and epilepsy; (3) class II & III; and (4) pregnancy and lactation.

The patients were assigned to one of three groups based on the number of LLLT sessions provided to patients, as follows: Group I (one session/week), group II (two sessions/week), and Group III (three session/week) The session continuous for 4 weeks. All patients were treated by the same practitioner. All patients received laser therapy using a 940-nm diode laser (EPICTM, BIOLASE, www.biolase.com) with power output at 1 W continuous wave. The energy density delivered to each trigger point was 4 J/cm². An adjustable handpiece was placed perpendicular to the site to create a spot size of 15 mm with slight pressure on the target point and irradiated for 60 s.

The laser handpiece was calibrated each time and protective eyewear was used by the patient and practitioner.

Measurements were taken before and after the laser therapy at (1, 2, 3, 4, and 8 weeks) to record the level of pain experienced during palpation and/or unassisted MMO, and the overall quality of life by a blind assessor.

The pain intensity was measured on a scale of 0 to 10, where 0 is no pain and 10 is the highest level of pain. The assessment of unassisted MMO (interincisal distance) involved measuring the distance between the upper and lower central incisors [8]. The quality of life was measured using the Oral Health Impact Profile questionnaire (OHIP-14) [17, 18], which consisted of 14 questions established into seven oral health sectors. The participant was asked to provide a rating for each question from 1 to 5 [never (score 0), scarcely ever (score 1), occasionally (score 2), somewhat frequently (score 3), and very often (scoring 4), and the total score was recorded which could range from 0 to 56 units. The higher scores indicated a greater level of impairment in the quality of life.

The statistical analysis was conducted using R version 4.3.2. Categorical variables were summarized by presenting frequencies and percentages. Numerical data were presented as mean ± standard deviation (SD). one-way ANOVA was used for between-group comparisons and within-group analysis over time. Additionally, generalized estimating equations (GEE) were employed as a multivariate analysis to estimate the impact of laser session numbers. A *p*-value below 0.05 was considered statistically significant.

## Results

This study included 90 participants randomly allocated into three equal groups of 30 participants each. The mean age of patients was 35.9 ± 12.6 years. Females represented the majority (83.3%) of participants. the baseline characteristics of age, sex, pain score, maximal mouth opening (MMO), and (OHIP-14) were not statistically significantly different between the three groups. (Table 1)

**Table 1:** Summary statistics of demographic data

Variables	Group I (*N* = 30)	Group II (*N* = 30)	Group III (*N* = 30)	F	*p*
Sex (*N*%)					0.5^a^
Female	24(80%)	27(90%)	24(80%)		
Male	6(20%)	3(10%)	6(20%)		
Age Mean ± SD	34.83 ± 12.43	34.33 ± 13.36	38.57 ± 12.01	1.01	0.37

F: One way ANOVA with (2,87) degree of freedom, a: Chi-square test, *p-value < 0.05

**Fig. 2:**
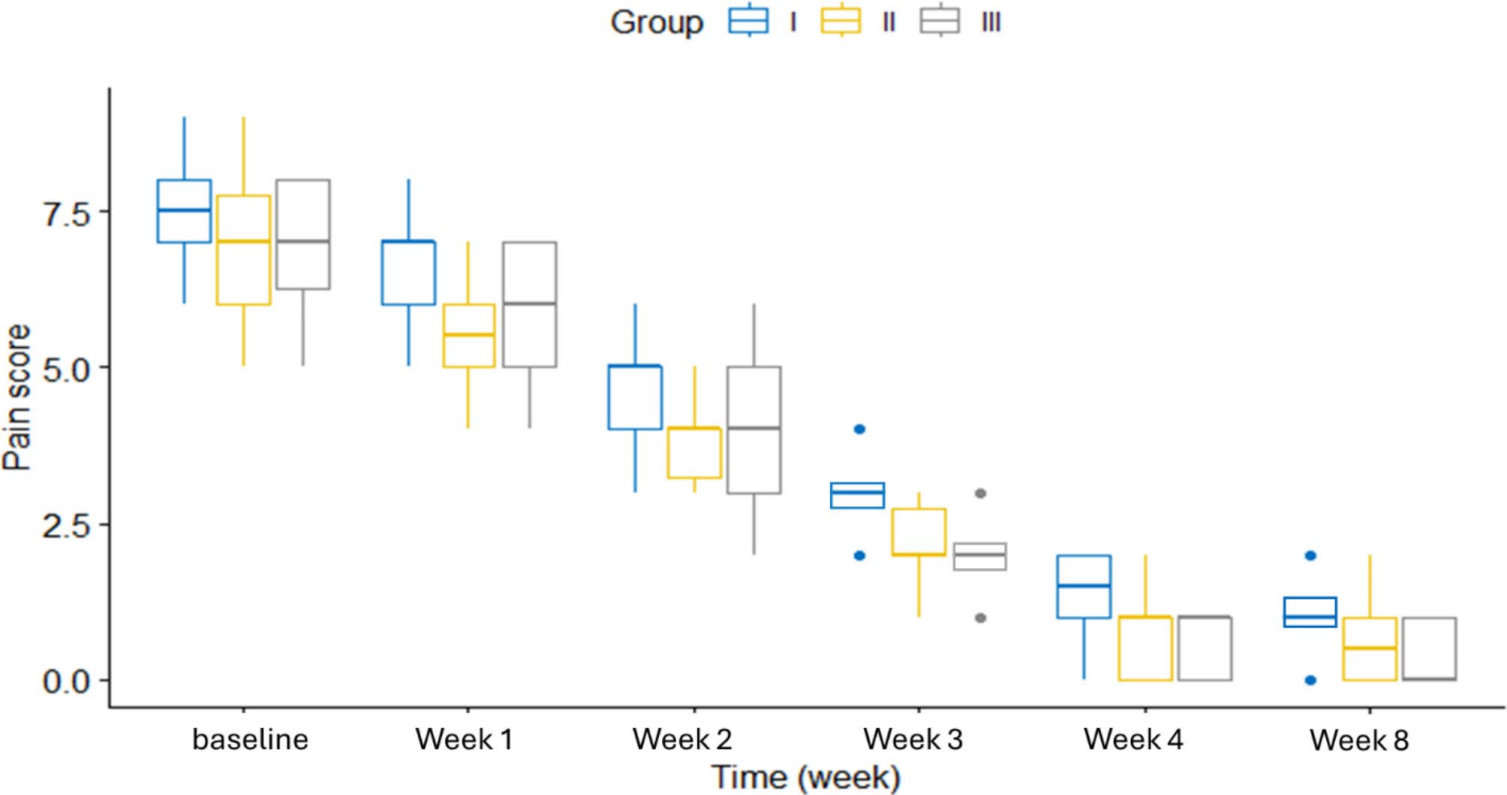
Pain score vs time in weeks in study groups (*N* = 90)

**Fig. 3:**
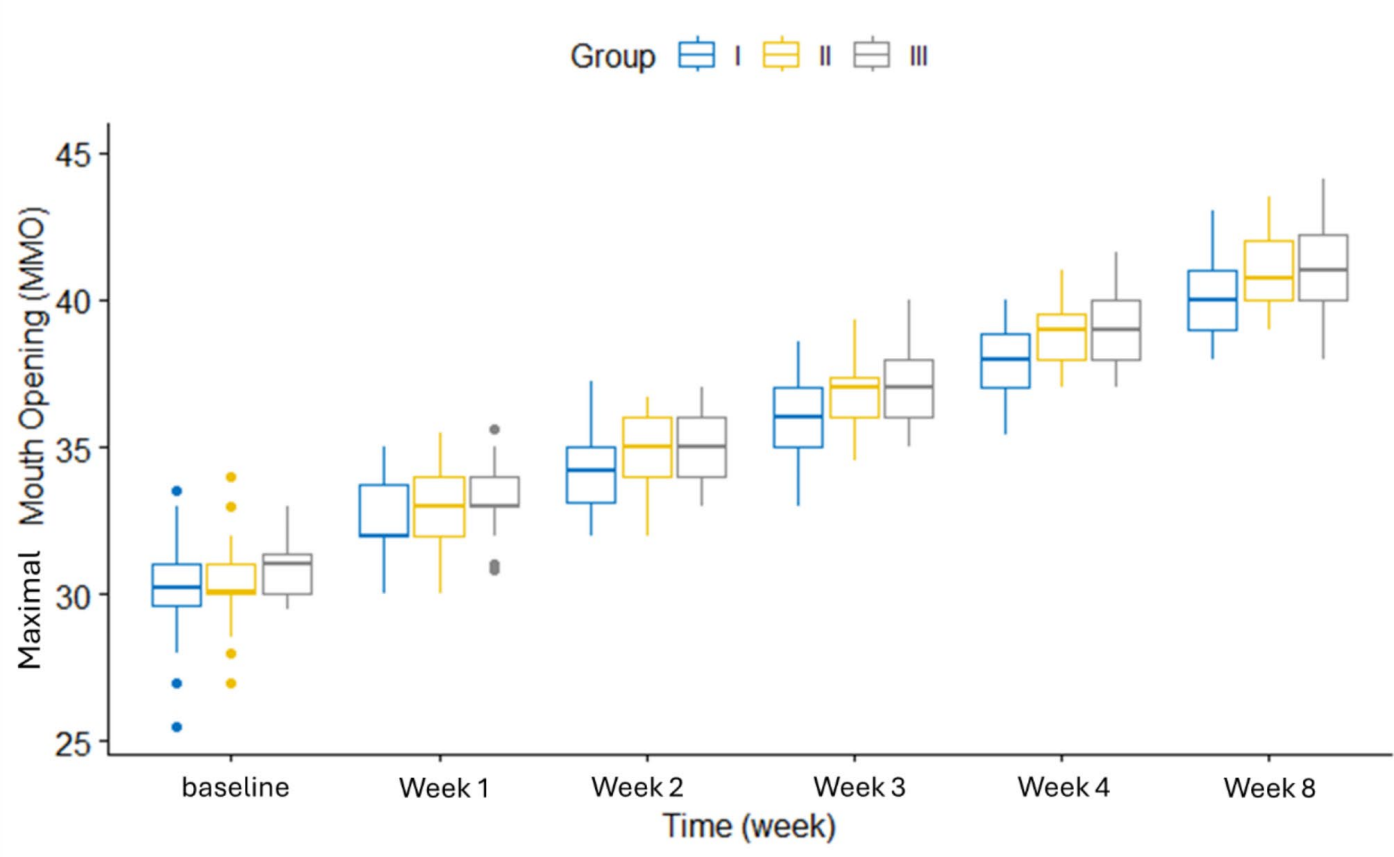
MMO vs time in weeks in study groups (*N* = 90)

**Fig. 4:**
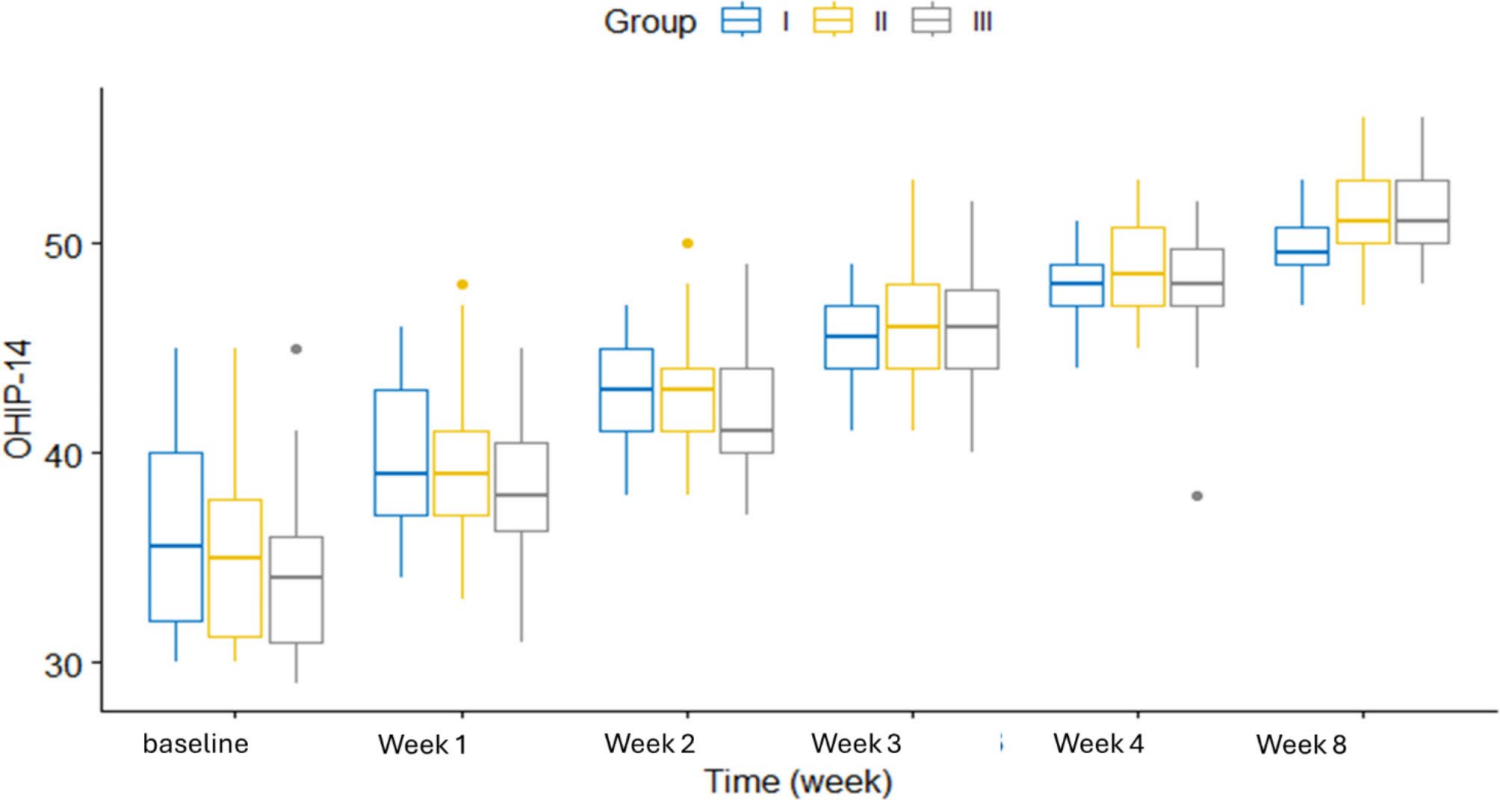
OHIP-14 vs time in weeks in study groups (*N* = 90)

The pain scores showed a highly statistically significant difference between the three groups (*p* < 0.001) over the 8-week study period. Figure 2 shows the changes in pain scores over time for each group. MMO was statistically significantly different between groups at week 4 and week 8. Figure 3 displays the MMO changes at different time points for each group. OHIP-14 score was statistically significantly different between groups at week 8. Figure 4 shows the OHIP-14 score changes over time in the three groups.

The highly significant effect of time on the study outcomes within each group. Pain scores decreased significantly over time in all three groups (*p* < 0.001). In group I, pairwise comparisons revealed significant differences between all time points. In groups II and III, all time points were significantly different except between pain at week 4 and week 8. Maximal mouth opening (MMO) significantly improved from baseline to week 8 in all groups. (OHIP-14) scores also demonstrated significant improvement over time within each group (Table 2).

**Table 2:** Longitudinal changes in outcomes within each group (within group analysis, *N* = 90)

Variable	Group	baseline	week 1	week 2	week 3	week 4	week 8	F	*p*
pain score	I	7.5 ± 0.97	6.53 ± 0.94	4.8 ± 0.89	3.03 ± 0.62	1.47 ± 0.57	1.07 ± 0.37	557.78	< 0.001*
	II	6.93 ± 0.94	5.57 ± 0.82	3.9 ± 0.66	2.03 ± 0.72	0.6 ± 0.56	0.53 ± 0.57	593.31	< 0.001*
	III	7 ± 0.91	5.87 ± 1.01	3.97 ± 1.03	2.07 ± 0.52	0.63 ± 0.49	0.43 ± 0.5	616.80	< 0.001*
MMO	I	30.3 ± 1.77	32.51 ± 1.29	34.38 ± 1.43	36.17 ± 2.27	37.83 ± 1.2	40 ± 1.23	271.70	< 0.001*
	II	30.47 ± 1.45	32.95 ± 1.42	34.84 ± 1.08	36.82 ± 1.05	38.83 ± 1.09	40.89 ± 1.32	624.76	< 0.001*
	III	30.89 ± 0.92	33.24 ± 1.14	35.06 ± 0.98	37.09 ± 1.26	39.09 ± 1.3	41.09 ± 1.59	581.61	< 0.001*
OHIP-14	I	36.23 ± 4.57	40.13 ± 3.55	42.97 ± 2.58	45.43 ± 2.1	47.77 ± 1.7	49.87 ± 1.48	230.15	< 0.001*
	II	35.13 ± 3.96	39.47 ± 3.44	42.8 ± 2.94	45.8 ± 2.78	48.97 ± 2.39	51.43 ± 2.24	324.47	< 0.001*
	III	34.3 ± 3.99	38.57 ± 3.66	42.3 ± 3.39	45.87 ± 2.71	47.9 ± 2.56	51.43 ± 1.92	361.08	< 0.001*

F: One-way repeated ANOVA, * *p*-value < 0.05

**Fig. 5:**
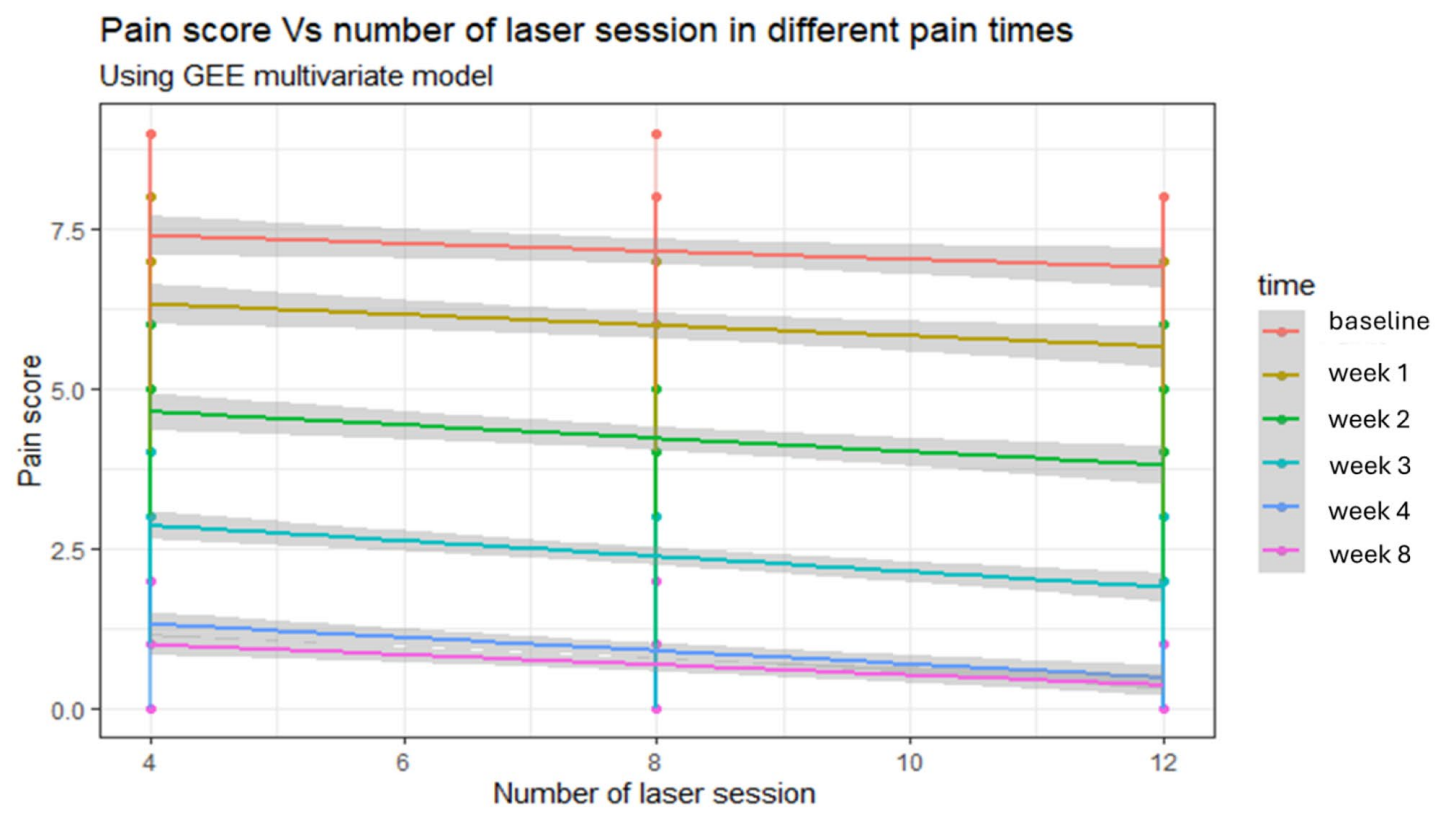
Pain score vs a number of laser session in different pain time using GEE model

GEE analysis assessed the effects of several laser sessions and time on pain scores. Increasing the number of laser sessions by 1 per week was associated with a 0.09 decrease in pain score (95% CI -0.11, -0.07; *p* < 0.001) Table 3. Figure 5 illustrates the relationship between pain score, number of laser sessions, and time. In summary, GEE analysis demonstrated that increasing the number of laser sessions and time points significantly impacted pain scores in the hypothesized directions.

**Table 3:** Estimate of no. Of laser sessions and time on pain score using GEE model (*N* = 90)

Variables	Estimate	95%CI	SE	*p*	WR^2^
(Intercept)	4.29	(4.12,4.46)	0.09	< 0.001*	0.101
no. of laser sessions	-0.09	(-0.11, -0.07)	0.01	< 0.001*	
Time: (Ref = baseline)					
week 1	3.59	(3.42,3.76)	0.09	< 0.001*	
week 2	2.44	(2.26,2.61)	0.09	< 0.001*	
week 3	0.67	(0.51,0.83)	0.08	< 0.001*	
week 4	-1.17	(-1.3,-1.04)	0.07	< 0.001*	
week 8	-2.65	(-2.77, -2.53)	0.06	< 0.001*	

CI: confidence interval, SE: stander error, WR^2^ : Weighted R2, * *p*-value < 0.05

## Discussion

Myofascial pain dysfunction syndrome is a neuromuscular disorder characterized by pain, muscle spasms, and muscle band contractions, which manifest as trigger points, commonly affecting the masseter muscles in the orofacial region [5, 7, 19]. LLLT had analgesic, healing, and anti-inflammatory effects on irradiated tissues that can positively affect the management of myofascial pain and dysfunctions [9–12].

The current study aimed to examine how varying the number of LLLT sessions could affect pain levels, MMO, and overall quality of life in patients diagnosed with masseter muscle trigger points. 90 patients were randomly allocated into three equal groups (*n* = 30) with 83.3% of the participants were Females. The null hypothesis of the study, which posited hat increasing the number of LLLT treatment sessions would not be associated with improved pain levels, MMO, and OHIP-14 was rejected. The higher ratio of female to male patients (three to five-fold higher female than male patients) can be attributed to lower pain tolerance, increased treatment demands, and psychological disorders among females [20–22].

The effects of time and laser treatment sessions on the pain score were evaluated using GEE approach. GEE is a statistical method with a member of the class of semiparametric regression techniques. This approach was used to study the interaction effects of various parameters on the treatment groups at various time points. It revealed robust, vigorous qualities during parameter estimation and is less susceptible to the variance structure specification. Due to its higher statistical power, smaller sample sizes, and fewer repeated measures required, GEE is more effective than the repeated measures analysis of variance (RM-ANOVA) method. RM-ANOVA primarily focuses on determining the significance of interactions and examining the magnitude of parameter estimates. Furthermore, a particular distribution of the outcome variable is not necessary for GEE to function. GEE is beneficial in studies with skewed data or small sample size which is difficult to validate the distribution [23, 24].

Pain is the major reason for patients with myofascial dysfunction syndrome to seek treatment. Pain occurred any TMD and pain reduction contributes to ameliorating the patient’s restricted jaw movement and masticatory function. Pain intensity was the primary outcome of the current study. As per study results, the pain scores showed a highly statistically significant difference between the three groups (*p* < 0.001) over the 8-week study period. This could be attributed to that, LLLT can induce photochemical reactions in the target cells. The main chromophore that absorbs red light is Cytochrome c, on the mitochondria of cells, this can organize the nitric oxide activity, ATP, calcium ions, and reactive oxygen species. Cytochrome c releases nitric oxide, increases the synthesis of ATP, and decreases oxidative stress in hypoxic or stressed cells subjected to laser therapy with great effect on tissue repair, cellular respiration, vasodilatation, increased pain threshold, and reduced inflammation [25, 26].

The Study results coincide with Ahrari et al. [27], Maia et al. [28], and Kulekcioglu et al. [29] who reported the optimal efficacy of LLLT for pain management. In contrast, Cuhna et al. [30], and Emshoff et al. [31] mentioned that LLLT has no therapeutic effect in MPDS-related pain and dysfunction.

Throughout the study period, the MMO showed improvement which can be related to pain intensity reduction. Khalighi et al. [15] studied the efficacies of LLLT and laser acupuncture therapy in MPDS treatment and concluded that MMO opening had been improved after LLLT.

Quality of life is a crucial factor to be considered when analyzing the effect of different chronic pain management strategies. One such tool for evaluating oral health-related quality of life and function is the OHIP-14. In the current study, follow-up evaluations of all groups showed improvement in the OHIP-14 score; which can be explained by decreasing the pain intensity and increasing the function [8].

The results of the present study showed that increasing the number of laser sessions by 1 per week was associated with a 0.09 decrease in pain score. This could be explained by the increased dose and duration of radiation. This is in line with Zhang et al. [12] who find that increased heterogeneity in the results, is considered of the study population and laser parameters. Sensitivity analysis revealed that the studies with larger doses and longer LLLT applications had more significant results although the large variation in laser dosage used across studies, it is difficult to draw firm conclusions about effective dosage from these studies.

The present study had several limitations. First, it did not compare the effects of different wavelengths of LLLT. Second, objective methods of assessment such as EMG could not be used due to limited resources.

## Conclusion

The results of the current study suggested that an increased number of LLLT sessions can reduce the pain and improve the MMO with great improvement in the life quality of the patients treated from the myofascial trigger point of the masseter muscle. Further studies comparing the effects of different wavelengths of LLLT and functional methods of assessments are recommended.

## Data Availability

The datasets used and/or analysed during the current study are available from the corresponding author upon reasonable request.

## Abbreviations

LLLT: Low-level laser therapy
MMO: Maximum mouth opening
OHIP-14: Oral Health Impact Profile-14 :
TMD: Temporomandibular disorder
GEE: Generalized estimating equations
MPDS-related pain: Myofascial pain dysfunction syndrome
EMG: Electromyography
